# Signalling strength determines proapoptotic functions of STING

**DOI:** 10.1038/s41467-017-00573-w

**Published:** 2017-09-05

**Authors:** Muhammet F. Gulen, Ute Koch, Simone M. Haag, Fabian Schuler, Lionel Apetoh, Andreas Villunger, Freddy Radtke, Andrea Ablasser

**Affiliations:** 1 Global Health Institute, Ecole Polytechnique Fédérale de Lausanne (EPFL), Station 19, 1015 Lausanne, Switzerland; 2 Swiss Institute for Experimental Cancer Research (ISREC), Ecole Polytechnique Fédérale de Lausanne (EPFL), Station 19, 1015 Lausanne, Switzerland; 3 Division of Developmental Immunology, Biocenter, Medical University of Innsbruck, Innrain 80-82, 6020 Innsbruck, Austria; 4 INSERM U866, Faculté de Médecine, Université de Bourgogne, 7 Boulevard Jeanne d’Arc, 21078 Dijon, France; 5 Tyrolean Cancer Research Institute, Innrain 66, 6020 Innsbruck, Austria

## Abstract

Mammalian cells use cytosolic nucleic acid receptors to detect pathogens and other stress signals. In innate immune cells the presence of cytosolic DNA is sensed by the cGAS–STING signalling pathway, which initiates a gene expression programme linked to cellular activation and cytokine production. Whether the outcome of the STING response varies between distinct cell types remains largely unknown. Here we show that T cells exhibit an intensified STING response, which leads to the expression of a distinct set of genes and results in the induction of apoptosis. Of note, this proapoptotic STING response is still functional in cancerous T cells and delivery of small molecule STING agonists prevents in vivo growth of T-cell-derived tumours independent of its adjuvant activity. Our results demonstrate how the magnitude of STING signalling can shape distinct effector responses, which may permit for cell type-adjusted behaviours towards endogenous or exogenous insults.

## Abstract (web summary)

The cGAS/STING signalling pathway is responsible for sensing intracellular DNA and activating downstream inflammatory genes. Here the authors show mouse primary T cells and T leukaemia are hyperresponsive to STING agonist, and this strong STING signalling is associated with apoptosis induction.

## Introduction

A fundamental feature of the innate immune system is the use of nucleic acid (NA) receptors as sensors for virus infection. In the cytosol of mammalian cells the best-characterised NA driven signal transduction pathways are the RIG-I like receptor (RLR) and cGAS–STING pathways, which detect RNA and DNA species, respectively^1^. Although RLRs and cGAS/STING have specificities for distinct ligands, both pathways engage a similar set of transcription factors, which coordinate the expression of type I interferons (IFN) and other antiviral and proinflammatory genes. Although traditionally viewed as a central part of the innate immune system, the expression of NA sensors is not restricted to professional antigen-presenting cells. Instead, RLRs and cGAS/STING are present in many mammalian cells. While much has been learned about the function of NA sensors in innate immune cells, less is known about their effector functions in other cell types. Identifying the signalling outputs of NA sensors is critical to understanding how antiviral networks are integrated into the specific cellular context within which they operate.

The cytosolic recognition of double-stranded (ds) DNA through the cGAS–STING signalling pathway is crucial for the recognition of DNA viruses, but also other pathogens including retroviruses and intracellular bacteria. Upon binding cytosolic dsDNA, cGAS catalyses the synthesis of cyclic GMP-AMP (cGAMP 2ʹ3ʹ), which in turn engages STING as a second receptor^2–6^. After its activation STING recruits Tank binding kinase (TB﻿K1), which then phosphorylates STING, thereby rendering STING capable of interacting with Interferon regulatory factor 3 (IRF-3)^7^. Phosphorylation of IRF-3, again mediated by TBK1, results in IRF-3 dissociation form STING, self-dimerisation and consequently IRF-3 translocation into the nucleus to regulate gene expression. In addition to IRF-3, Nuclear Factor Kappa B﻿ (NF-κB) is also a key element within the STING signalling cascade. The coordinated activation of transcription factors promotes the induction of various antiviral genes, in particular type I IFNs and IFN-stimulated genes (ISG). In addition, STING signalling is also associated with the production of many proinflammatory cytokines and chemokines.

Although the cGAS–STING signalling pathway is best characterised for generating an antiviral response, increasing evidence indicates that cGAS and STING are also involved in the regulation of alternative, non-inflammatory cellular responses. For example, evidence exists that STING promotes cross-presentation, triggers autophagy and, in some instances, induces cell death^8, 9–12^. While these reports highlight diverse, type I IFN-independent functions of STING, the regulation of those remains less well characterised.

Given their highly specific function in adaptive immunity, we decided to assess the response elicited by STING in T cells. Here, we show that T cells exhibit an alternate signalling outcome in response to small molecule STING agonists, which manifests in apoptosis rather than the production of type I IFNs. We find that the induction of apoptosis is due to high expression levels of STING in T cells, which triggers an intensified response that is associated with the induction of IRF-3-dependent and p53-dependent proapoptotic genes. Remarkably, this proapoptotic STING response is also functional in cancerous T cells. As such, pharmacological hyperactivation of STING prevents tumour growth of T-cell-derived cancers independent of its adjuvant activity. Together, our study uncovers the magnitude of STING signalling as a means through which STING generates proapoptotic effects and proposes to exploit this mechanism therapeutically in the context of T-cell-derived malignancies.

## Results

### Induction of a proapoptotic STING response in T cells

**Fig. 1:**
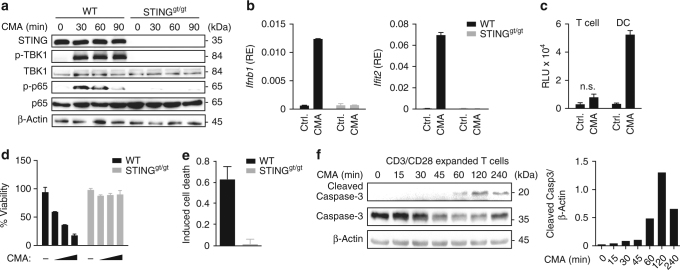
STING activation induces apoptosis in T cells. **a** CD4^+^ T cells from wild-type (WT) or STING^gt/gt^ mice were treated with DMSO or 10-carboxymethyl-9-acridanone (CMA) for the indicated times and phosphorylation of TBK1 and NF-κB (p65) was assessed by immunoblot. **b** CD4^+^ T cells from WT or STING^gt/gt^ mice were treated with DMSO or CMA for 4 h and abundance of mRNAs of the indicated genes was measured by RT-qPCR. **c** Supernatants from CD4^+^ T cells or dendritic cells (DC) treated with DMSO or CMA were collected after 16 h. A bioassay was used to measure the production of type I IFNs (RLU relative light units). **d**, **e** CD4^+^ T cells from WT and STING^gt/gt^ mice were treated with DMSO or CMA. After 16 h cell viability **d** and percentage of apoptotic cells **e**, assessed by Annexin V staining, were determined. **f** WT CD4^+^ T cells were stimulated with CMA for the indicated times and caspases-3 cleavage was assessed by immunoblot. Quantification of cleaved Caspase-3 relative to β-Actin levels is shown on the right panel. Data are representative of *n* = 3 (**a**, **f**) independent experiments or mean and s.d. of technical replicates (*n* = 2) of one representative experiment out of *n* = 3 (**b**, **d**, **e**) or *n* = 2 (**c**) independent experiments are shown. Unprocessed original blots are shown in Supplementary Fig. 9.

To determine the effect of STING activation in primary T cells, we used fluorescence-activated cell sorting (FACS) to isolate highly pure primary CD4^+^ T cells (hereafter referred to as T cells) from the spleens of wild-type (WT) mice and mice lacking STING (Goldenticket (STING^gt/gt^))^13^. We found that STING was expressed at high levels in splenic T cells (Fig. 1a). To circumvent unspecific effects of liposomes on the activation status of T cells, we employed the cell permeable small molecule STING agonist 10-carboxymethyl-9-acridanone (CMA) for stimulation experiments^14^. Short-term treatment of undifferentiated CD4^+^ T cells with CMA resulted in phosphorylation of TBK1 and NF-κB p65 in WT cells but not in cells from STING^gt/gt^ mice (Fig. 1a). CMA elicited similar activities in in vitro differentiated Th1, Th2 and Th17 cells (Supplementary Fig. 1a). Thus, STING is active in T cells. STING signalling is best known to trigger the secretion of cytokines, especially type I IFNs and to induce the expression of ISGs. We therefore assessed the upregulation of type I IFNs (*Ifnb1*) and *Ifit2* in T cells. CMA-treated T-cells induced expression of *Ifnb1* and *Ifit2* on mRNA level, however production of type I IFNs on protein level was not detectable when assessed by an IFN bioassay (Fig. 1b, c). Instead, when cultured in the presence of CMA overnight, T cells displayed visible signs of cell death (Fig. 1d). Similar to undifferentiated CD4^+^ T cells, CD8^+^ T cells and distinct in vitro differentiated T-cell subsets committed to cell death upon CMA treatment (Supplementary Fig. 1b, c). Further analysis of the mechanism of CMA-induced cell death revealed that T cells from WT mice, but not from STING^gt/gt^ mice, displayed Annexin V binding on their cell surface and triggered activation of caspases-3/7, both hallmarks of apoptotic cell death (Fig. 1e, f and Supplementary Fig. 2). These data indicate that the STING signalling pathway is functional in primary T cells, yet instead of triggering type I IFN production it results in the induction of apoptosis.

### Cell type-specific apoptosis induction on STING activation

**Fig. 2:**
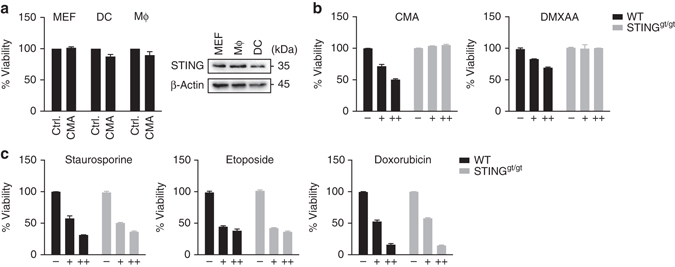
STING activation induces apoptosis in a cell type-specific manner. **a** Left: Primary mouse embryonic fibroblasts (MEF), bone marrow-derived macrophages (MΦ) and bone marrow-derived DCs (DC) were treated with DMSO or 10-carboxymethyl-9-acridanone (CMA) and 16 h later cell viability was determined. Right: STING expression in distinct cell types was analysed by immunoblot. **b**, **c** CD4^+^ T cells from wild-type (WT) and STING^gt/gt^ mice were left untreated or stimulated with CMA (0.125 mg/ml, 0.25 mg/ml), DMXAA (10 µg/ml, 100 µg/ml), staurosporine (100 nM, 500 nM), etoposide (1 µg/ml, 5 µg/ml) or doxorubicin (0.4 µg/ml, 1 µg/ml) overnight. Cell viability was assessed by CellTiter Blue assay. Representative results from *n* = 3 independent experiments (**a**; right panel) or mean and s.d. of technical replicates of one representative experiment out of *n* = 3 experiments are shown **a**–**c**. Unprocessed original blots are shown in Supplementary Fig. 9

Numerous studies have so far described the role of STING in antiviral gene expression, but only very few reports have proposed a link between STING and cell death. This led us to speculate that the cell death-inducing capability of STING might not be a universal signalling output, but that it would rather represent a specific effect, differentially controlled among distinct cell types. Indeed, when other cell types including primary mouse embryonic fibroblasts (MEFs), primary bone marrow-derived dendritic cells (BMDCs) or macrophages (BMDMs) were examined for apoptosis induction no overt cell death response was observed (Fig. 2a). From these results we conclude, that the induction of apoptosis is not a general response to the activation of STING, but instead it is a cell type-specific phenomenon that is evident in primary T cells but not in MEFs, BMDCs or BMDMs. We do note, however, that additional cell populations may exist that exhibit a different or intermediate cell death response to STING agonists compared to the clear dichotomy described above. Similar to CMA, the small molecule STING activator DMXAA-stimulated apoptosis in T cells (Fig. 2b). In contrast to the STING-dependent effects of CMA and DMXAA, the presence of STING did not affect the cell death response of staurosporine, etoposide or doxorubcine (Fig. 2c). Thus, the apoptosis-inducing capability of STING is not a preset phenomenon, but instead it is evident in T cells upon small molecule activation of the STING pathway.

### Involvement of BH3-only proteins in STING-mediated apoptosis

**Fig. 3:**
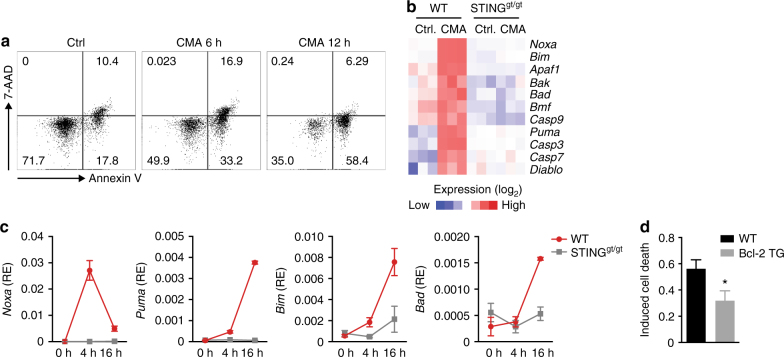
BH3-only proteins are involved in STING-triggered T cell apoptosis. **a** Flow cytometric analysis of apoptosis of CD4^+^ T cells left untreated or treated with 10-carboxymethyl-9-acridanone (CMA) for 6 h and 12 h, assessed by Annexin V and 7-AAD staining. Numbers in quadrants present percentages. **b** Heat-map of RNA-sequencing analysis. Genes related to cell intrinsic apoptosis with statistically significant increase in CMA-treated over DMSO-treated CD4^+^ T cells are shown (*n* = 3 biological replicates; *P* < 0.05; Student’s *t*-test). **c** T cells from wild-type (WT) or STING^gt/gt^ mice were untreated or stimulated with CMA for 4 h and 16 h. Abundance of mRNAs for the indicated genes was quantified by RT-qPCR. **d** CD4^+^ T cells of the genotypes indicated were treated with DMSO or CMA. After 16 h the percentage of apoptotic cells was assessed by Annexin V and 7-AAD staining. Data are representative of *n* = 3 independent experiments (**a**) or mean and s.d. of technical replicates of one representative experiment out of *n* = 3 (**c**) or *n* = 2 (**d**) independent experiments are shown. *P < 0.05; (Student’s *t*-test)

We next sought to understand how apoptosis is regulated in T cells in response to STING activation. Multiple mechanisms of apoptosis initiation have been described, some of which depend on stimulus-driven assembly and activation of protein complexes, while others are controlled through the transcriptional induction of proapoptotic genes, usually belonging to the BCL-2 family of proteins. When analysing T cells committing to apoptosis, we observed a gradual increase of T-cell apoptosis over time after CMA treatment, rather than a short burst of cell death (Fig. 3a). Given these kinetics, we hypothesised the involvement of de novo gene expression in the apoptosis process. Gene expression profiling of CMA-stimulated T cells revealed the proapoptotic BH3-only protein *Pmaip1* (also known as *Noxa*) in the list of the most strongly induced genes (Fig. 3b). We also observed increased expression of the BH3-only proteins *Bbc3* (*Puma*) and *Bcl2l11* (*Bim*) and transcripts encoding other apoptotic proteins including *Smac/Diablo*, *Casp3*, *Casp7* and *Apaf1* (Fig. 3b). Direct measurement of mRNA abundance in T cells treated with CMA validated the STING-dependent upregulation of the BH3-only proteins *Noxa*, *Puma*, *Bim* and *Bad* (Fig. 3c). Since *Noxa* and *Puma* were markedly induced already at 4 h post stimulation, we considered these transcripts as reliable markers for triggering apoptosis in CMA-stimulated T cells. BH3-only proteins carry out their proapoptotic function by neutralising prosurvival BCL-2 family proteins or direct activation of Bax and/or Bak^15^. To establish functional significance of BH3-only proteins in CMA-triggered apoptosis, we utilised T cells from BCL-2 transgenic mice (Bcl2-TG), in which the function of all BH3-only proteins listed above is compromised. CMA-triggered apoptosis was significantly reduced in T cells constitutively expressing BCL-2 supporting the contribution of BH3-only proteins in STING-dependent cell death (Fig. 3d).

### IRF-3 and p53 regulate the proapoptotic programme

**Fig. 4:**
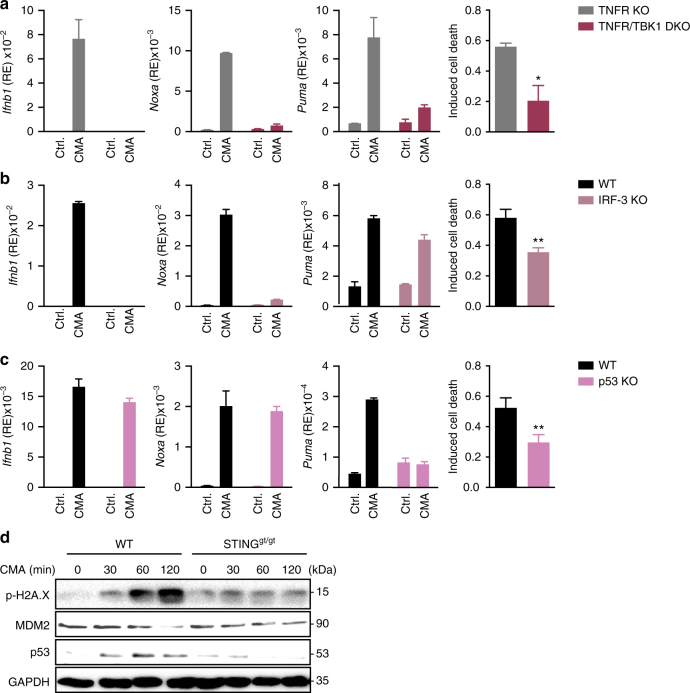
Cooperation of IRF-3 and p53 for the STING-mediated proapoptotic transcriptional programme. **a**–**c** CD4^+^ T cells of the indicated genotypes were treated with DMSO or 10-carboxymethyl-9-acridanone (CMA). After 16 h the percentage of apoptotic cells was assessed by Annexin V and 7-AAD staining (right) or upregulation of depicted genes was determined by RT-qPCR (left). **d** CD4^+^ T cells from wild-type (WT) or STING^gt/gt^ mice were stimulated with CMA for the indicated times and p53, MDM2 and phosphorylation of Histone H2A.X were assessed by immunoblot. Data are representative of *n* = 2 **d** independent experiments or mean and s.d. of technical replicates of one representative experiment out of *n* = 3 independent experiments are shown **a**–**c**. *P < 0.05 and **P < 0.01 (Student’s *t*-test). Unprocessed original blots are shown in Supplementary Fig. 9

The prototypical transcriptional programme elicited by STING in macrophages or DCs is largely dependent on IRF-3 and NF-κB p65, which are both activated by TBK1^7^. To address whether apoptosis of T cells is also dependent on these factors, we examined T cells lacking TBK1 or IRF-3. In response to CMA T cells derived from TNFR/TBK1 DKO or IRF-3 KO mice were unable to upregulate *Ifnb1* and *Noxa* expression compared to respective controls (Fig. 4a, b and Supplementary Fig. 3a). However, although *Puma* expression was reduced in the absence of TBK1, its expression was not affected by loss of IRF-3 (Fig. 4b). Direct measurement of apoptosis also revealed differences between TBK1 and IRF-3 deficiency. Whereas absence of TBK1 considerably affected induction of apoptosis following CMA treatment, absence of IRF-3 had only a partial effect (Fig. 4a, b). Thus, IRF-3 is a central mediator of STING-dependent activities in T cells, yet it does not account for all its transcriptional responses. Because p53 (encoded by *Trp53* in mice) represents one of the most prominent regulators of *Puma*, we examined the CMA response of T cells from p53-deficient mice. Whereas absence of p53 did not alter the induction of *Ifnb1* or *Noxa*, it abrogated the increased expression of *Puma* (Fig. 4c). Similar to IRF-3 deficient cells, T cells from p53 deficient mice exhibited a partially reduced apoptosis response (Fig. 4c). Consistent with an involvement of p53, T cells displayed upregulation of p53 following stimulation by CMA (Fig. 4d). Further analysis revealed that CMA promoted phosphorylation of γH2AX, a hallmark of DNA damage, and led to the degradation of MDM2 (Fig. 4d and Supplementary Fig. 3b). Taken together, these results indicate that the coordinated action of IRF-3 and p53 is responsible to induce STING-mediated T-cell apoptosis by promoting the upregulation of *Noxa* and *Puma*.

Type I IFNs are well known to exert anti-proliferative effects. Although the observations described above point towards cell-autonomous induction of apoptosis, it may be possible that type I IFNs reinforce the proapoptotic function of CMA-triggered cell death. To address this, we incubated T cells with CMA in the presence or absence of an antibody that antagonises the type I IFN receptor. In addition, we added recombinant IFN-β to T cells stimulated with CMA. Of note, neither blocking nor augmenting type I IFN signalling affected the expression of proapoptotic genes or influenced overall cell viability (Supplementary Fig. 3c, d). These data therefore indicate that type I IFNs are not majorly contributing to CMA-triggered T-cell death.

### An intensified STING response induces apoptosis

**Fig. 5:**
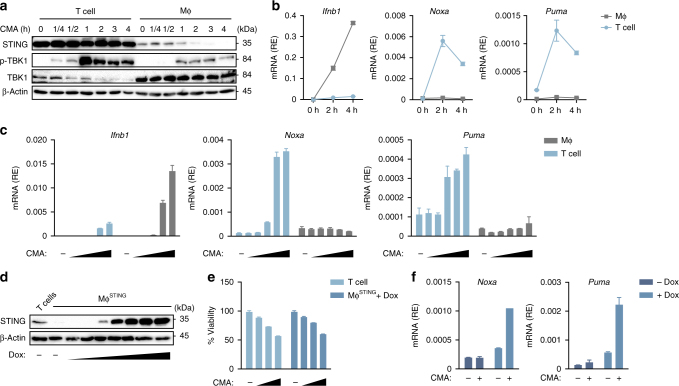
Intensified STING signalling leads to apoptosis in T cells. **a** Wild-type (WT) CD4^+^ T cells and bone marrow-derived macrophages (MΦ) were left untreated or stimulated with 10-carboxymethyl-9-acridanone (CMA) for the indicated time points and immunoblot analysis was performed with indicated antibodies. **b** WT CD4^+^ T cells and macrophages were stimulated with CMA for 2 h and 4 h, followed by quantification of abundance of mRNAs for the indicated genes by RT-qPCR. **c** WT CD4^+^ T cells and macrophages were stimulated with increasing doses of CMA (31.25–250 μg/ml) and upregulation of depicted genes was determined by RT-qPCR. **d** Macrophages transduced with a doxycycline-inducible murine STING construct (MΦ^STING^) were treated with doxycycline and STING expression was determined by immunoblot. **e** WT CD4^+^ T cells and MΦ^STING^ were treated with CMA and cell viability was assessed by CellTiter Blue assay. **f** MΦ^STING^ in the presence or absence of doxycycline were treated with CMA and proapoptotic gene expression was assessed by RT-qPCR. Data are representative of three independent experiments **a**, **d** or mean and s.d. of technical replicates of one representative experiment out of *n* = 3 independent experiments are shown **b**, **c**, **e**, **f**. Unprocessed original blots are shown in Supplementary Fig. 9

The observation that despite sharing the same signalling pathway, macrophages or DCs failed to induce expression of proapoptotic transcripts and, therefore, did not commit to apoptosis has implications for the mechanism underlying the T-cell-selective response (Supplementary Fig. 4a). We hypothesised that either the signalling magnitude or its duration might differ in between the distinct cell types. To test these possibilities we compared the expression levels of STING between T cells and macrophages over time. Surprisingly, we noted substantially higher amounts of STING protein in T cells compared to macrophages (Fig. 5a). In line with previous reports, STING was rapidly degraded in macrophages following its activation, whereas in T cells STING expression was less affected even at 4 h post stimulation (Fig. 5a)^9^. We next monitored the kinetics of STING signalling by measuring phosphorylation of TBK1, the most upstream event in the signal transduction pathway. Whereas T cells showed rapid and strong phosphorylation of TBK1 after stimulation, this occurred with delayed kinetics and less overall magnitude in macrophages (Fig. 5a). In contrast, the decay of TBK1 phosphorylation proceeded in T cells at a time scale close to the time scale in macrophages, indicating that the signalling durations are similar. We next compared gene expression in T cells and macrophages following treatment with CMA. Interestingly, despite strong upregulation of *Ifnb1*, macrophages failed to induce *Noxa* and *Puma*, whereas both proapoptotic genes were readily induced in T cells reaching a maximum as early as 2 h post stimulation (Fig. 5b). The induction of proapoptotic genes in T cells followed a similar dose-dependency as the induction of *Ifnb1* in T cells and macrophages (Fig. 5c). This indicates that the differential outputs between these cell types are not due to differences in overall sensitivity, but rather a consequence of a qualitative distinct signalling mechanism.

To further substantiate the possibility that the intensity of STING signalling determines the cellular outcome, we generated a macrophage cell line stably expressing a doxycycline (Dox)-inducible construct of murine STING (MΦ^STING^). Treatment with Dox elicited a dose-dependent augmentation of the expression levels of STING (Fig. 5d). Remarkably, increasing STING levels equivalent to those of T cells was sufficient to render MΦ^STING^ susceptible towards CMA-induced cell death (Fig. 5e and Supplementary Fig. 4b). Furthermore, examining CMA-induced expression of *Noxa* and *Puma* in MΦ^STING^ revealed strong induction of both transcripts in the presence of Dox (Fig. 5f). Taken together, this indicates that upon CMA treatment T cells exhibit an intensified signalling response, which allows for the expression of proapoptotic transcripts and, eventually, apoptosis induction. In support of this, macrophages expressing a hyperactive version of murine STING MΦ^STING V154M^ committed to cell death in the absence of an additional STING ligand (Supplementary Fig. 4c–e)^16, 17^.

### Antitumour property of STING in T-cell-derived malignancies

**Fig. 6:**
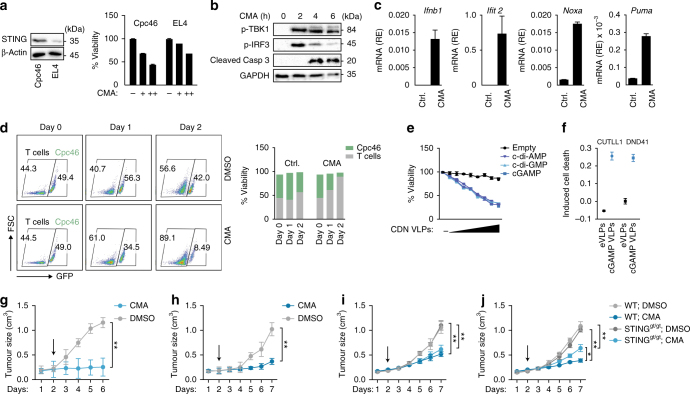
Pharmacological hyperactivation of STING promotes apoptosis of T-cell-derived malignant cells. **a** Murine T-ALL cells (Cpc46) and lymphoma cells (EL4) were incubated with 10-carboxymethyl-9-acridanone (CMA) for 16 h and cell viability was assessed by CellTiter Blue assay. **b** Cpc46 cells were treated with DMSO (upper panel) or CMA (lower panel) and phosphorylation of TBK1, IRF-3 and cleavage of caspase-3 was assessed at indicated times by immunoblot. **c** Cpc46 cells were treated with DMSO or CMA and the induction of indicated genes was quantified by RT-qPCR after 4 h. **d** Primary CD4^+^ T cells (in the presence of CD3 and CD28 activation) and Cpc46 cells (*x* axis, GFP^+^ cells) were cultured in a 1:1 ratio and treated with DMSO (upper panel) or CMA (lower panel). After 1 day and 2 days percentages of GFP^+^ cells (Cpc46 cells) vs. GFP^−^ primary T cells were determined by FACS. **e** Cpc46 cells were incubated with empty viral particles (eVLPs) or VLPs containing cGAMP (cGAMP VLPs), c-di-AMP or c-di-GMP for 16 h and cell viability was assessed by CellTiter Blue assay. **f** CUTLL1 cells and DND41 cells were left untreated or incubated with eVLPs or cGAMP VLPs and percentages of apoptotic cells were measured by FACS after 16 h. **g**–**j** Subcutaneous in vivo growth of Cpc46 cells (**g**) and EL4 cells (**h**) in Rag2^−/−^γc^−/−^ mice or Cpc46 cells (**i**) and EL4 cells (**j**) in wild-type (WT) or STING^gt/gt^ mice that were treated with or without (Ctrl.; treatment with diluent only) CMA (500 µg), daily after tumour size reached up to 200 mm^3^. (Cpc46, *n* = 3 mice per treatment group; EL4, *n = *5 mice per treatment group). Tumour size was measured daily. Arrow indicates starting point of the treatment. Data are representative of *n* = 3 (**a**; left) or *n* = 2 (**b**) independent experiments or mean and s.d. of one representative experiment out of *n* = 3 (**a**, **c**–**f**) or mean and s.d of *n* = 3 (Cpc46 cells) or *n* = 5 (EL4 cells) mice per treatment group are shown **g**–**j**. **P < 0.01 (two-way ANOVA). Unprocessed original blots are shown in Supplementary Fig. 9

On the basis of these data we then explored whether targeted ‘hyperactivation’ of STING may offer the possibility to kill malignant T cells. Towards this end a panel of T-cell acute lymphoblastic leukaemia (T-ALL) cells obtained from NOTCH1-driven mouse models and EL4 T-cell lymphoma cells were subjected to treatment with CMA^18^. Within all cell lines examined, CMA induced a strong reduction in cell viability (Fig. 6a and Supplementary Fig. 5a). Interestingly, the amount of STING protein present correlated well with the ensuing cell death response (Fig. 6a). Similar to primary T cells, CMA-treated T-ALL cells (Cpc46) committed to apoptosis as monitored by Annexin V staining and caspases-3 cleavage (Fig. 6b and Supplementary Fig. 5b). Moreover, activation of STING in Cpc46 cells led to rapid phosphorylation of TBK1 and IRF-3 and strong upregulation of *Ifnb1*, *Ifit2*, *Noxa* and *Puma* (Fig. 6b, c). Interestingly, the cytotoxic effects of CMA were even more pronounced in Cpc46 cells compared to primary T cells (Fig. 6d). Thus, malignant T cells reveal a high degree of vulnerability towards the death inducing properties of STING.

Besides species-specific, small molecule STING activators, cyclic dinucleotides (CDNs) are potent, natural activators of STING and trigger potent type I IFN responses^19^. We thus examined the proapoptotic activity of CDNs in T cells. However, addition of cGAMP to T cells led to only modest activation of apoptosis. We reasoned that this could be due to the inefficient up-take of cGAMP into T cells yielding only suboptimal STING activation. To overcome this obstacle, we tested viral particles (VLPs) as delivery vehicles for CDNs, a method described to mediate transfer of cGAMP in between cells^20, 21^. cGAMP containing VLPs (cGAMP VLPs), but not empty VLPs (eVLPs), elicited robust activation of STING in T cells, as monitored by upregulation of STING-dependent gene expression and apoptosis induction (Supplementary Fig. 6a–e). We then returned to Cpc46 cells and tested the activity of CDN-containing VLPs to trigger tumour cell death. Notably, infecting Cpc46 cells with cGAMP VLPs led to a strong reduction of the live cell population (Fig. 6e). VLPs incorporating cyclic di-AMP or cyclic di-GMP had an equivalent activity with regards to cell death induction (Fig. 6e). Using the VLP-based approach to target STING in human T-cell lymphoma cells (CUTLL-1) and human T-ALL cells (DND41), we observed significantly reduced viability of both human cell lines via induction of apoptosis (Fig. 6f). Finally, we examined whether pharmacological activation of STING displays direct antitumour activity in vivo. Immunocompromised Rag2^−/−^γc^−/−^ mice (lacking T cells, B cells and NK cells) were injected subcutaneously with Cpc46 cells or EL4 cells. Upon tumour establishment (~200 mm^3^) mice were subjected to daily treatments with CMA or vehicle control administered by intratumoral injection. Whereas tumours grew continuously in mice receiving vehicle only, tumour growth was either completely prevented in the setting of T-ALL (Cpc46) or markedly reduced in the setting of T-cell lymphoma (EL4) (Fig. 6g, h). Following CMA injections, both Cpc46-derived and EL4-derived tumours displayed an increase in apoptotic cells (Supplementary Fig. 7). To further substantiate that tumour cell intrinsic mechanisms mediate the antitumour properties of CMA, we transplanted Cpc46 and EL4 cells into STING^gt/gt^ mice and assessed changes in tumour volumes upon CMA treatment. Importantly, intratumoral injection of CMA prevented tumour cell growth in both WT and STING^gt/gt^ mice, thus indicating that the antitumour properties of CMA rely on the activation of STING within tumour cell (Fig. 6i, j). Although the improved efficacy of CMA in WT mice indicates that activation of STING in host cells can further augment tumour cell clearance. In sum, we conclude that in the context of T-cell-derived malignancies, STING agonists unfold antitumour properties in an adjuvant-independent manner.

## Discussion

PRRs are best characterised for their function in promoting innate immunity upon encountering microbe-associated or danger-associated molecular patterns. The intracellular recognition of DNA through the cGAS–STING pathway is pivotal for host defence against a variety of distinct pathogens^22, 23^. Both cGAS and STING were originally described as key molecules to promote type I IFN induction by DCs or macrophages upon infection with DNA viruses^2, 24^. These discoveries established that innate immune cells, including professional antigen-presenting cells, but also endothelial cells or fibroblasts, use cGAS and STING to sense pathogens and to initiate an antiviral immune response. However, in contrast to other PRR systems, cGAS and STING are present in cells from various distinct origins. Here we report that overt stimulation of the STING pathway triggers apoptosis in primary and malignant T cells, which may be utilised therapeutically.

Type I IFNs are signature cytokines produced by many cells in response to various STING agonists, such as the cGAS enzymatic product cGAMP as well as bacterial CDNs or small molecule agonists^3, 5, 6, 14, 25^. Although activation of STING in T cells led to upregulation of antiviral genes, the most prominent effect observed was the initiation of apoptosis. This is in sharp contrast to the response elicited by STING activators in macrophages, DCs or MEFs, which we found to be largely apoptosis-resistant. Similar to T cells, a previous study has reported that primary and malignant B cells commit to cell death in response to STING agonists^12^. Although the underlying mechanism of cell death induction in B cells differs from the one reported here, it is interesting to note that the induction of apoptosis appears to be a common effector response of the STING pathway in lymphocytes. More generally, we conclude that the cellular context considerably affects the choice of the effector programme that is triggered by STING (Supplementary Fig. 8).

Connecting the activation of STING to T-cell apoptosis may offer key advantages for the host in certain contexts. As such, given that T cells are not part of the innate immune system they might not be competent for initiating extracellular antiviral functions. Indeed, type I IFNs are generally regarded to be very low expressed in T cells. Thus, from the host perspective, apoptosis may represent the ‘best’ choice to eliminate infected T cells. On the other hand, calibrating the STING signalling output allows ‘professional’ innate immune cells to remain functionally active while coping with pathogens.

Mechanistically, the cell type-specific differences can be explained by our observation that T cells exhibit increased expression levels of STING and thereby display an intensified STING response relative to macrophages or DCs. This ‘overshooting’ signalling response activates p53, which acts together with IRF-3 to promote the apoptosis programme. Consistent with this model of intensified STING signalling, the apoptosis-resistant phenotype of macrophages could be overcome by either altering the ‘concentration’ of STING or by expressing a hyperactive version of STING. It will be important to further characterise the means by which STING levels are controlled in T cells.

The ability of cells to commit to apoptosis strongly correlated with the induction of the two BH3-only proteins Noxa and Puma, which are controlled by IRF-3 and p53 independently of each other. The functional importance of the involvement of BH3-only proteins was best revealed by experiments with cells derived from Bcl2-TG mice, which globally antagonises the action of BH3-only proteins. As a result, even though STING-dependent gene expression is not altered in Bcl2-TG mice, the amount of T cells undergoing apoptosis is markedly reduced. The lack of upregulation of both *Noxa* and *Puma* in apoptosis-resistant macrophages or DCs further supports a function of these factors during STING-mediated apoptosis. TBK1 is required for STING-dependent type I IFN induction and, up until now, considered as the most upstream kinase in the STING signal transduction pathway. Our data revealed that TBK1 is also involved in the T-cell intrinsic apoptotic programme, illustrated by the rescue of cell death and by the impairment of *Noxa* and *Puma* induction in TBK1-deficient T cells. Thus, our data suggest that TBK1 acts as a core regulator of the proapoptotic transcriptional programme downstream of STING. Further studies are warranted for the elucidation of the precise molecular connection between STING/TBK1 and p53.

Beyond delineating a cell type-specific regulation of the STING signalling pathway, the study presented here has also important therapeutic implications. As such, we noted that the phenomenon of STING-mediated apoptosis could be extended towards malignant T cells as occurring in the setting of T-ALL or T-cell lymphoma. In this regards, the observation that T-cell-derived tumour cells are even more sensitive towards treatment with STING ligands is especially intriguing. Our data suggest that within T-ALL cells the underlining signal transduction pathway is identical to the one described for primary T cells, involving both *Noxa* and *Puma* upregulation. Thus, even though it is likely that mutations within p53 could reduce the efficacy of STING agonists to trigger the apoptosis pathway in cancer cells, these mutations account for only a minor percentage in T-ALL. For the future, it will be interesting to investigate whether the STING-dependent apoptosis pathway can be exploited in additional cancer cell types of hematopoietic or other origin.

Given their potent immunostimulatory effect, STING agonists have attracted a lot of attention to boost antitumour immune responses. For example, it was shown that intratumoral injection of STING ligands induced regression of established tumours by generating systemic antitumour immune responses^8, 26, 27^. Our data indicate that depending on the cellular origin of the tumour, STING ligands may also exert direct antitumour properties—independently from their adjuvant activity. We also assessed VLPs as vehicles to deliver CDNs to malignant T-cell lines and thereby revealed impressive activation of cell death in both murine as well as human cancer cell lines. Based on this, we speculate that CDN containing VLPs generated to specifically target cancer cells might be a promising strategy to deliver CDNs directly to cancer cells. This might be especially relevant in the context of T-ALL, where tumour cells reside in areas not accessible for intratumoral drug delivery.

Importantly, our findings are not in controversy to the capability of cGAMP or other STING agonists as adjuvant for immunotherapy. In fact, when added to T cells cGAMP was only weak in triggering apoptosis. Instead, the data presented here reveal a cell-specific phenomenon, which is evident upon strong stimulus delivery. In line with this notion, T cells isolated from STING-associated vasculopathy with onset in infancy (SAVI) patients, who bear hyperactive mutations of STING, showed an increased apoptotic phenotype^16^. Besides T cells also monocytes and endothelial cells from SAVI patients were positive for apoptotic cell markers^16^. It is possible that the cell type-specific, proapoptotic pathway that described here may also contribute to the disease manifestation of SAVI patients.

Finally, it remains open whether our observation of STING-mediated cell death may also be naturally relevant during infection with viruses, which have a tropism for T cells. Several studies have detailed the involvement of cGAS/STING during infection of DCs and monocytes with retroviruses, such as human immunodeficiency virus-1 (HIV-1), HIV-2 or human T-cell leukaemia virus type 1 (HTLV-1)^11, 28, 29^. Future studies are needed to explore the role of T-cell intrinsic STING-driven apoptosis in this context.

In summary, we show that the outcome of (cGAS–) STING signalling is strongly influenced by the cell type under study and we propose that STING-mediated stimulation of apoptosis should be further explored as treatment option in the context of T-cell-derived human malignancies.

## Methods

### Mice

Wild-type mice (Stock#: 000664) and *STING* ^*gt/gt*^ mice^13^ (Goldenticket) (Stock#: 017537) and Rag2^−/−^γc^−/−^ mice (Stock#: 014593) were purchased from Jackson Laboratories. Mice on C57BL/6 background were bred and maintained under specific pathogen-free (SPF) conditions at Ecole Polytechnique Federale de Lausanne (EPFL), Switzerland. Mice (8–12 weeks) from the same gender were used for all experiments. Mice were sacrificed in a CO_2_ chamber followed by cervical dislocation. These animal experiments were approved by the Service de la consommation et des affaires vétérinaires (1066 Épalinges, Canton of Vaud, Switzerland). *Tnf* ^−/−^ and *Tnf* ^−/−^ *Tbk1* ^−/−^ mice^30^ were generated by Shizuo Akira (Osaka University, Japan) and spleens from these mice were obtained from Christine Möser (Goethe University, Germany).

### Primary cells and cell lines

Mouse CD4^+^ T cells were isolated from single-cell suspensions from spleens and lymph nodes by negative selection using the CD4 T-cell isolation kit (Miltenyi Biotech, #130-104-454) according to the manufacturer’s instructions. In some experiments CD4^+^CD44^lo^ cells were sorted from single-cell suspensions from spleens and lymph nodes by flow cytometry. For anti-CD3 and anti-CD28-stimulated differentiation, naïve sorted CD4^+^CD44^lo^ cells were activated with plate-bound anti-CD3 (Clone: 145-2C11) (3 μg/ml) and anti-CD28 (Clone: 37.51) (3 μg/ml) (both eBioscience) and in the presence of 5 ng/ml TGF-β and 10 ng/ml IL-6 and 5 mg/ml anti-IFN-γ (XMG 1.2) (for T_H_17), 10 ng/ml rm-IL-4 and 100U rm-IL-2 and 5 mg/ml anti-IFN-γ (XMG 1.2) (for T_H_2), 10 ng/ml rm-IL-12 (for T_H_1) (Immunotools). For stimulation murine T cells were plated in 96-well plates at 5 × 10^6^ cells/ml. BMDMs were generated by culturing bone marrow cells with L929-cell-conditioned medium (LCCM) (10%) in DMEM medium for 6 days. BMDCs were generated by culturing bone marrow cells in IMDM medium supplemented with 10% (v/v) FCS, Ciprofloxacin (Bayer Schering Pharma) and the cytokines GM-CSF (20 ng/ml) and IL-4 (20 ng/ml) (both Immunotools) for 6 days. For stimulation, BMDMs and BMDCs were plated in 96-well plates at 1 × 10^6^ cells/ml. Primary MEFs and immortalised mouse macrophages (kindly provided by Fabio Martinon (University of Lausanne, Switzerland)) were cultured in DMEM (Life Technologies) supplemented with 10% (v/v) FCS and Ciprofloxacin (Bayer Schering Pharma). CUTLL1 (a kind gift from the laboratory of Adolfo Ferrando, Columbia University, USA) and DND41 (DSMZ-No: ACC-525; DSMZ cell repository, Braunschweig, Germany) were cultured in RPMI-1640 (Life Technologies) containing 20 mM HEPES, 10% (v/v) FCS and Ciprofloxacin. Cells were repeatedly tested for mycoplasma using specific primers. No method of cell line authentication was used.

For the isolation of human CD4^+^ T cells, PBMCs were obtained by Ficoll—Hypaque density gradient centrifugation, and CD4^+^ cells were isolated by MACS using the CD4^+^ cell isolation kit (Miltenyi Biotech, #130-096-533)) according to the manufacturer’s instructions. Human CD4^+^ T cells were then activated and expanded with 2.5 μg/ml PHA-L and 100 U IL-2 (both Sigma). The studies using primary human cells were approved by the Commission Cantonale d‘Èthique de la Recherche sur l’Etre Humain, Lausanne, Switzerland.

### Reagents and constructs

CMA (Cat.#: 17927) was obtained from Sigma. pEFBOS-based expression plasmids for a cyclic di-GMP synthase, for a cyclic di-AMP synthase (DacA), for murine cGAS and murine mCherry-tagged STING were previously described^5, 31, 32^. Lentiviral vectors encoding for mCherry-tagged STING and GFP are based on pTRIPZ.

### Cell stimulation

If not indicated otherwise cells were stimulated with CMA at a final concentration of 125 μg/ml or treated with 1 µl of concentrated cGAMP-VLPs. RNA expression was analysed after 4 h, whereas cell viability and apoptosis was analysed after overnight treatment. Supernatants for type I IFN bioassay were collected after 24 h. Cell lysates for Immunoblot were collected as indicated.

### Flow cytometry

To sort CD4^+^ T cells from the spleen cell surface staining was performed in PBS, 1% BSA (Sigma), 1 mM EDTA (AppliChem). Sorting was performed on MoFlo Astrios. Annexin V binding buffer, fluorochrome-conjugated Annexin V and 7-AAD were purchased from Biolegend and used according to manufacturer’s instructions. Briefly, cells were washed once with PBS and once with Annexin V binding buffer. Cells were stained with Annexin V (1:200 dilution) for 15 min at 4 °C in Annexin V binding buffer. Data were acquired on an Accuri C6 (BD) flow cytometer and analysed in FlowJo. The induced cell death was calculated as [(% death with CMA treatment − % death without CMA treatment)/(100 − % death without CMA treatment)]. The FACS gating strategies are shown in Supplementary Fig. 10.

### Gene expression profiling

For RNA sequencing naïve T cells were sorted from the spleens of wild-type mice and STING^gt/gt^ mice and expanded in the presence of anti-CD3 and anti-CD28 antibodies (eBioscience) for 2 days. Cells were rested 1 day in RPMI-1640 medium supplemented with 10% (v/v) FCS, 5% HEPES buffer (Amimed), 2% L-glutamine (Life Technologies) and Ciprofloxacin (Bayer Schering Pharma), without antibodies and stimulated with either DMSO or 125 μg/ml CMA overnight. Total RNA was isolated from the cells using the RNAeasy Mini Kit (Qiagen). RNA-seq libraries were prepared using 1000 ng of total RNA and the Illumina TruSeq Stranded mRNA reagents (Illumina) on a Sciclone liquid handling robot (PerkinElmer) using a PerkinElmer-developed automated script. Cluster generation was performed with the resulting libraries using the Illumina TruSeq SR Cluster Kit v4 reagents and sequenced on the Illumina HiSeq 2500 using TruSeq SBS Kit v4 reagents. Sequencing data were processed using the Illumina Pipeline Software version 1.82. Heat maps were produced from normalised expression data using Cluster 3.0 for computation and JTreeview for visualisation.

### Cell viability assay

Cells were plated in 96-wells and treated with DMSO or CMA overnight. Quantification of viable cells was performed using a CellTiter-Blue Cell Viability Assay (Promega, #G8090) according to the manufacturer’s instructions.

### Measurement of caspases-3/7 activity

Cells were plated in 96-wells and treated with DMSO or CMA. After incubation an equal amount of living cells were lysed and quantification of caspases-3/7 activity was performed using the Caspase-Glo 3/7 Assay (Promega) according to the manufacturer’s instructions.

### Type I IFN bioassay

Mouse type I IFN levels in the supernatants of stimulated T cells or BMDCs were determined by incubating LL171 cells stably expressing an ISRE-luciferase construct with the respective supernatants for 5 h. LL171 cells were lysed in passive lysis buffer (Promega) and luciferase activity was measured using luciferin as substrate.

### Immunoblotting

Cells were lysed in 1 × Laemmli buffer and denatured at 95 °C for 5 min. Cell lysates were separated by 10% SDS-PAGE and transferred onto nitrocellulose membranes. Blots were incubated with anti-STING (D2P2F), Phospho-TBK1 (D52C2), Phospho-NF-κB p65 (93H1), cleaved caspases-3 (Asp175) (5A1E), p53 (1C12) (all Cell Signaling) and anti-TBK1 (108A429) (Novus Biologicals) (all 1:1000 dilution). As a secondary antibody anti-rabbit-IgG-HRP (1:2000 dilution) (Santa Cruz Biotechnology) was used. Anti-β-actin-HRP or GAPDH (both 1:5000 dilution) was used as control. ECL signal was recorded on the ChemiDoc XRS Biorad Imager and data were analysed with Image Lab (Biorad).

### Production of lentiviral particles and CDN-containing VLPs

Lentiviral particles were produced by transfecting HEK293T cells (kind gift from Didier Trono, EPFL, Switzerland) with pCMVDR8.74 as packaging construct, pMD2.G for VSV-G pseudotyping and lentiviral vectors encoding for GFP-FLAG or for mCherry-tagged mouse STING as previously described^33^. Virus-containing cell supernatants were collected at 24, 36 and 48 h and filtrated over 0.2 µm filters. Subsequently supernatants were concentrated by ultracentrifugation and aliquots were stored at—80 °C. Immortalised murine macrophages were transduced with lentiviral particles at a MOI < 1. Stable mC-STING or GFP-expressing immortalised macrophages were generated under puromycin selection. For the production of cGAMP-containing virus like particles the protocol from above was applied expect that lentiviral vectors were replaced by eukaryotic expression vectors encoding for murine cGAS, cyclic di-GMP synthase, cyclic di-AMP synthase or GFP (as control).

### Quantitative RT-qPCR

Total RNA was isolated using the RNAeasy Mini Kit (Qiagen) and cDNA was synthesised using the RevertAid First Strand cDNA Synthesis kit (Fermentas). Quantitative RT-qPCR was performed in duplicate using Maxima SYBR Green Master Mix (Thermo Scientific) on an Applied Biosystems 7900HT machine. For all gene expression data beta-Actin was used as an endogenous normalisation control. Primer sequences are as follows: mmACTB forward, 5′-AGCCATGTACGTAGCCATCC-3′; mmACTB reverse, 5′-CTCTCAGCTGTGGTGGTGAA-3′; mmIFNB1 forward, 5′-CTCCAGCTCCAAGAAAGGAC-3′; mmIFNB1 reverse, 5′-TGGCAAAGGCAGTGTAACTC-3′; mmIFIT2 forward, 5′-GCTCTGGAAAAGGACCCGAA-3′; mmIFIT2 reverse, 5′-GCTTCAGTGCCAAGAGGACT-3′; mmPUMA forward, 5′-CAAGAAGAGCAGCATCGACA-3ʹ; mmPUMA reverse, 5ʹ-TAGTTGGGCTCCATTTCTGG-3ʹ; mmNOXA forward, 5ʹ-GGAGTGCACCGGACATAACT-3ʹ; mmNOXA reverse, 5ʹ-TTGAGCACTCGTCCTTCA-3ʹ; mmBIM forward, 5ʹ-GCCAAGCAACCTTCTGATGT-3ʹ; mmBIM reverse, 5ʹ-CTGTCTTGCGGTTCTGTCTG-3ʹ; mmBAD forward, 5ʹ-CGAAGGATGAGCGATGAGTT-3ʹ; and mmBAD reverse, 5ʹ-CCCACCAGGACTGGATAATG-3ʹ.

### In vivo subcutaneous tumour models

For tumour establishment, 5 × 10^6^ Cpc46 cells or 2.5 × 10^6^ EL4 cells were suspended in 100 μl Matrigel (Corning) and injected subcutaneously into 8–10 week old Rag2^−/−^γc^−/−^, wild-type mice or STING^gt/gt^ mice. Upon reaching a tumour size up to 0.2 cm^3^, mice were randomised into treatment groups and DMSO or CMA (500 μg/100 μl) were injected daily into the tumour. Measurements of tumours were performed daily using callipers. For the analysis of CMA-induced tumour cell death, tumours from mice were collected 1 day after DMSO or CMA injection. The tumours were dissociated in RPMI containing 80 μg/ml Liberase TL and 250 U/ml DNase I (Sigma) to obtain single cells for further analysis. No statistical methods have been used to predetermine sample size. No animals were excluded from the study. Experiments were conducted non-blinded.

### Statistical analysis

Statistical significance was calculated as described in the figure legends. Prism 6 software was used to generate graphs and to perform statistical analysis. *P* values of statistical significance are represented as **P < 0.01, *P < 0.05.

### Data availability

RNA sequencing data have been deposited in the Gene Expression Omnibus (GEO) under the accession code (GSE100411). All data available from the corresponding author on request.

## Electronic supplementary material

Supplementary Information
