# Supplementary material for: Signalling strength determines proapoptotic functions of STING
Source: Nat Commun. 2017 Sep 5;8:427. doi: 10.1038/s41467-017-00573-w (PMC5585373; doi:10.1038/s41467-017-00573-w)
Supplement: Supplementary file 1 — Supplementary Information [file 41467_2017_573_MOESM1_ESM.pdf]

Description of Supplementary Files

File name: Supplementary Information
Description: Supplementary figures.

Supplementary Figure 1

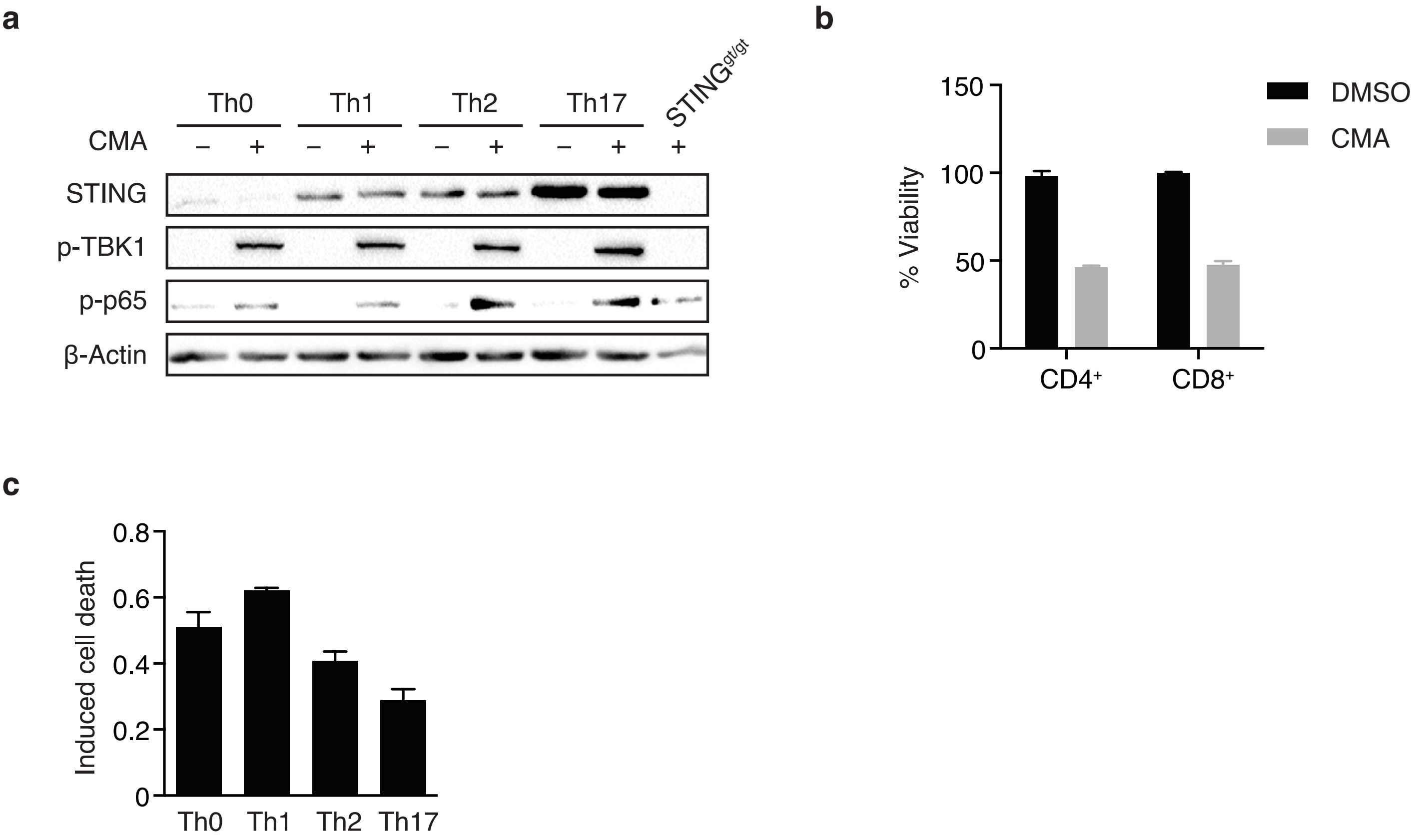

Supplementary Fig. 1 Activation of STING in distinct T helper cell subsets

(**a**) Immunoblot analysis of untreated or 10-carboxymethyl-9-acridanone (CMA)-stimulated wild-type T cells after 3 days of Th0, Th1, Th2 or Th17 differentiation of naïve T cells. Cells were rested in medium overnight and stimulated with 0.25 mg/ml CMA for 1 h. Th0 cells from STING$^{gt/gt}$ served as control. (**b**) CD4$^+$ or CD8$^+$ T cells isolated from the spleen of wild-type mice were stimulated with CMA (0.25 mg/ml) overnight. Cell viability was assessed by CellTiter Blue assay. (**c**) T cells were differentiated as in (**a**) and stimulated with 0.25 mg/ml CMA overnight and the percentage of apoptotic cells was assessed by Annexin V and 7-AAD staining. Mean and s.d. of technical replicates (n=2) are shown (**b**, **c**). Data are representative of n=2 independent experiments.

Supplementary Figure 2

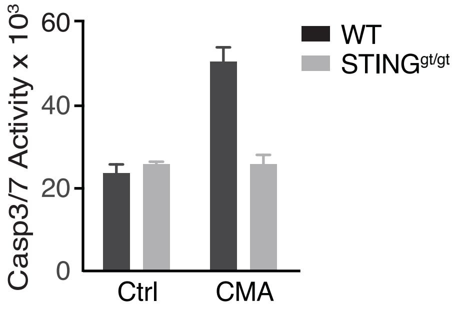

Supplementary Fig. 2 STING activation induces Caspase 3/7 activity in T cells
T cells from WT and STING$^{gt/gt}$ mice were stimulated with 10-carboxymethyl-9-acridanone (CMA) or left untreated. After 6 h caspases 3/7 activity was determined. Mean and s.d. of n=2 technical replicates of one representative experiment from n=2 independent experiments are shown.

Supplementary Figure 3

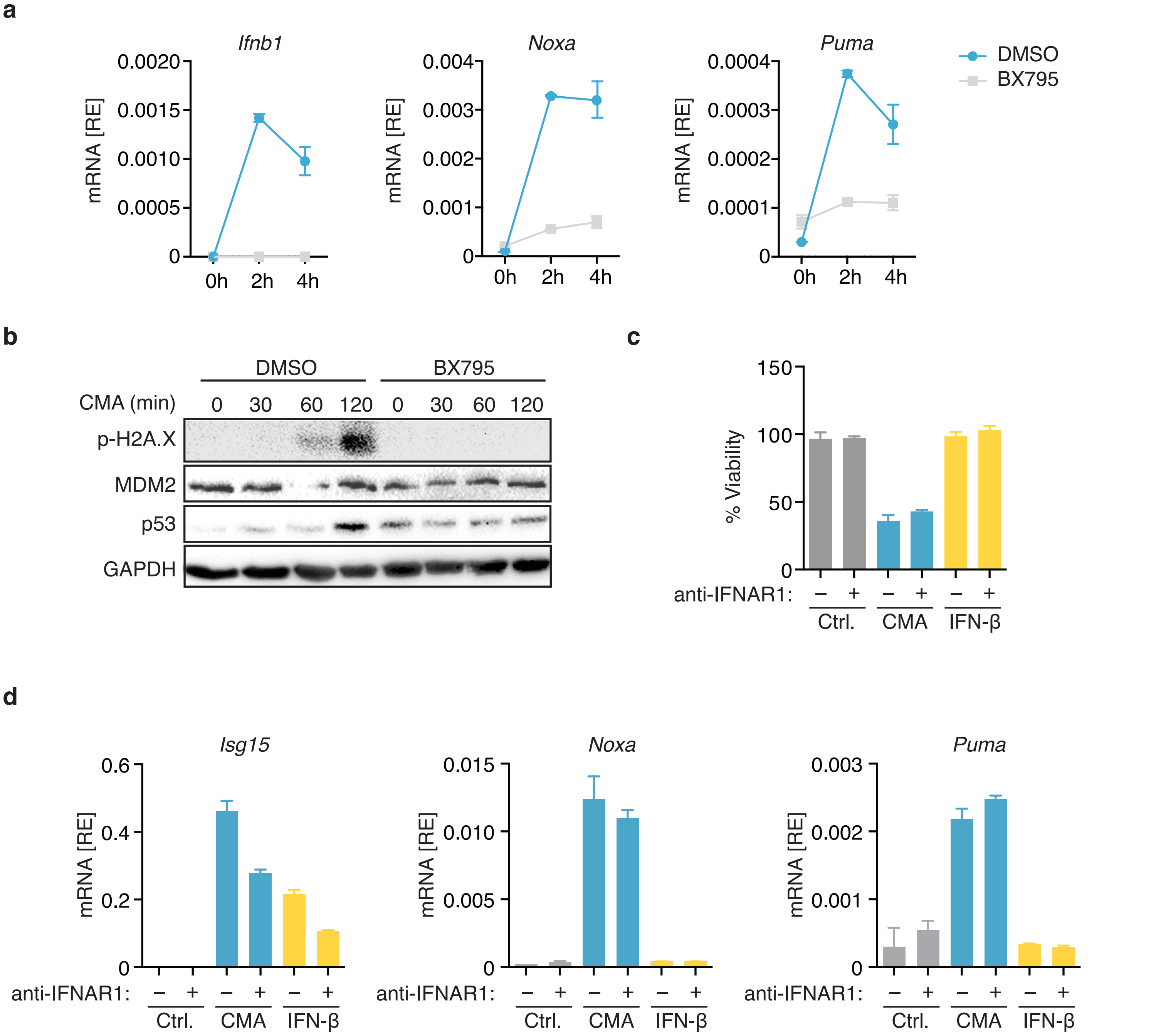

Supplementary Fig. 3 TBK1-dependent and IFN-β-independent regulation of proapoptotic genes in T cells
(**a**) CD4⁺ T cells were pretreated with the TBK1 inhibitor BX795 (2 μM) or DMSO for 30 min and then stimulated with 10-carboxymethyl-9-acridanone (CMA) (0.25 mg/ml) for the indicated time points. Gene expression was analyzed by RT-qPCR. (**b**) CD4⁺ T cells were pretreated with the TBK1 inhibitor BX795 (2 μM) or DMSO for 30 min and then stimulated with CMA (0.25 mg/ml) for the indicated time points. Activation of indicated signaling molecules was assessed by immunoblot. (**c**, **d**) CD4⁺ T cells were incubated with 10 μg/ml anti-IFNAR1 (MARI-5A3) and stimulated with CMA or 100 U recombinant IFN-β (Biolegend) or left untreated. After overnight incubation cell viability was assessed by CellTiter Blue assay (**c**) or after 4 h mRNA expression of depicted genes was examined by RT-qPCR (**d**). Data are representative for two independent experiments (**b**) or mean and s.d. of n=2 technical replicates from n=2 independent experiments are shown (**a**, **c**, **d**).

Supplementary Figure 4

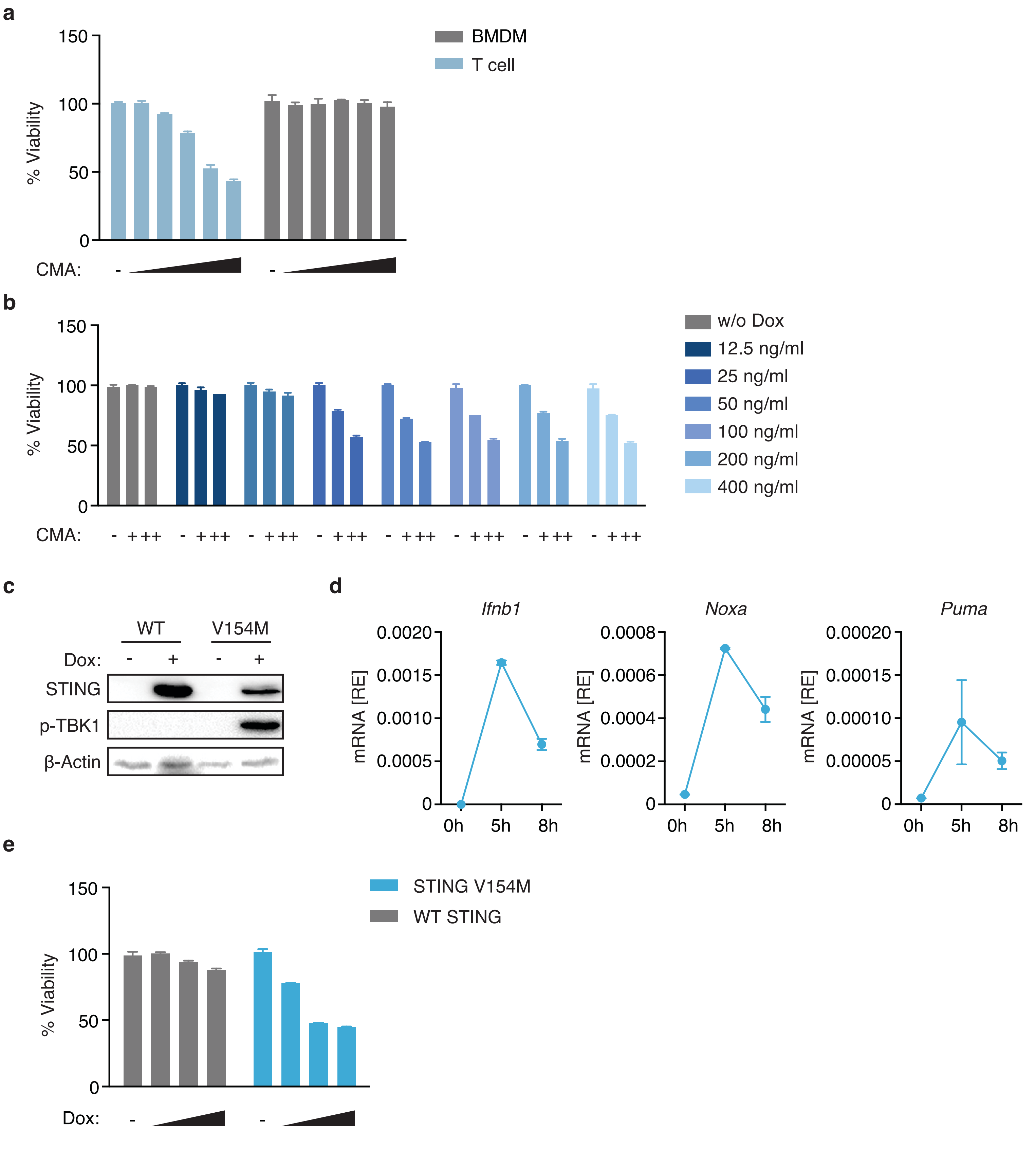

Supplementary Fig. 4 **Overexpression of STING renders macrophages susceptible to cell death**

(**a**) T cells and BMDMs were stimulated with 10-carboxymethyl-9-acridanone (CMA) (serial diluation from 0.5 μg/ml to 0.03125 μg/ml) or DMSO. After overnight incubation cell viability was assessed by CellTiter Blue Assay. (**b**) Immortalized macrophages transduced with an inducible construct for mouse STING were treated with doxycycline (Dox) as indicated for 5 h. Cells were then stimulated with CMA (0.25 μg/ml or 0.5 μg/ml) or DMSO overnight and survival of macrophages was assessed by CellTiter Blue assay. (**c**) Immortalized macrophages transduced with an inducible construct for mouse STING (WT) or for a hyperactive mutant version of mouse STING (V154M) were incubated with doxycycline (0.1 μg/ml) for 5 h. STING level and phosphorylation of TBK1 were assessed by immunoblot. (**d**) Macrophages expressing doxycycline-inducible V154M mmSTING were incubated with doxycyline (0.1 μg/ml) for 5 h or 8 h and expression of indicated genes was quantified by RT-qPCR. (**e**) Survival of macrophages was measured by using CellTiter Blue Assay after overnight incubation with increasing doses of doxycycline (concentrations: 50 ng/ml, 100 ng/ml, 200 ng/ml). Representative results from n=2 independent experiments are shown (**c**) or mean and s.d. of technical replicates (n=2) of one representative experiments of n=2 independent experiments are shown (**a**, **b**, **d**, **e**).

Supplementary Figure 5

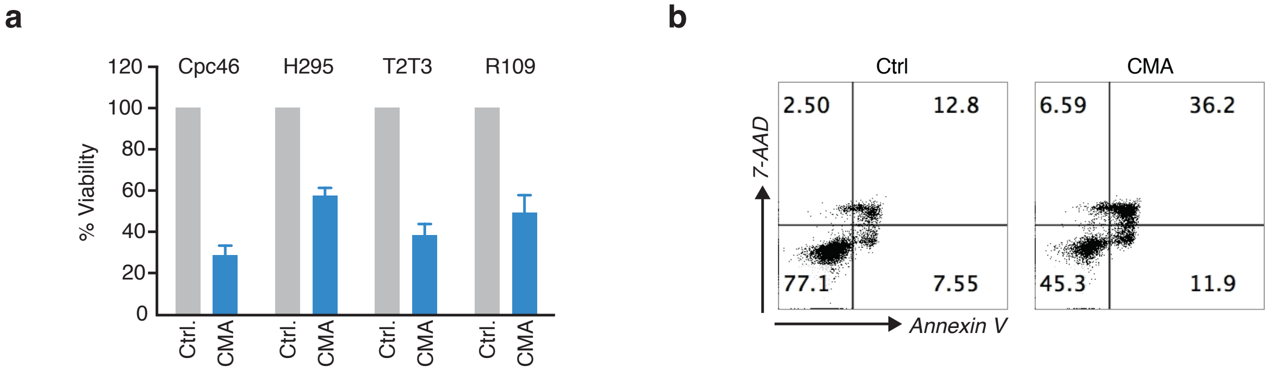

Supplementary Fig. 5 **Activation of STING in T cell-derived malignant cells induces apoptosis *in vitro***
(**a**) Various primary murine T-ALL cell lines were incubated with 10-carboxymethyl-9-acridanone (CMA) for 16 h and cell viability was assessed by CellTiter Blue assay. (**b**) T-ALL Cpc46 cells were treated with DMSO or stimulated with CMA and percentages of apoptotic cells were determined by flow cytometry analysis after 6 h using Anexin V and 7-AAD staining. Data are representative of three independent experiments (b) or mean and s.d. of technical replicates (n=2) of one representative experiment of n=3 independent experiments are shown.

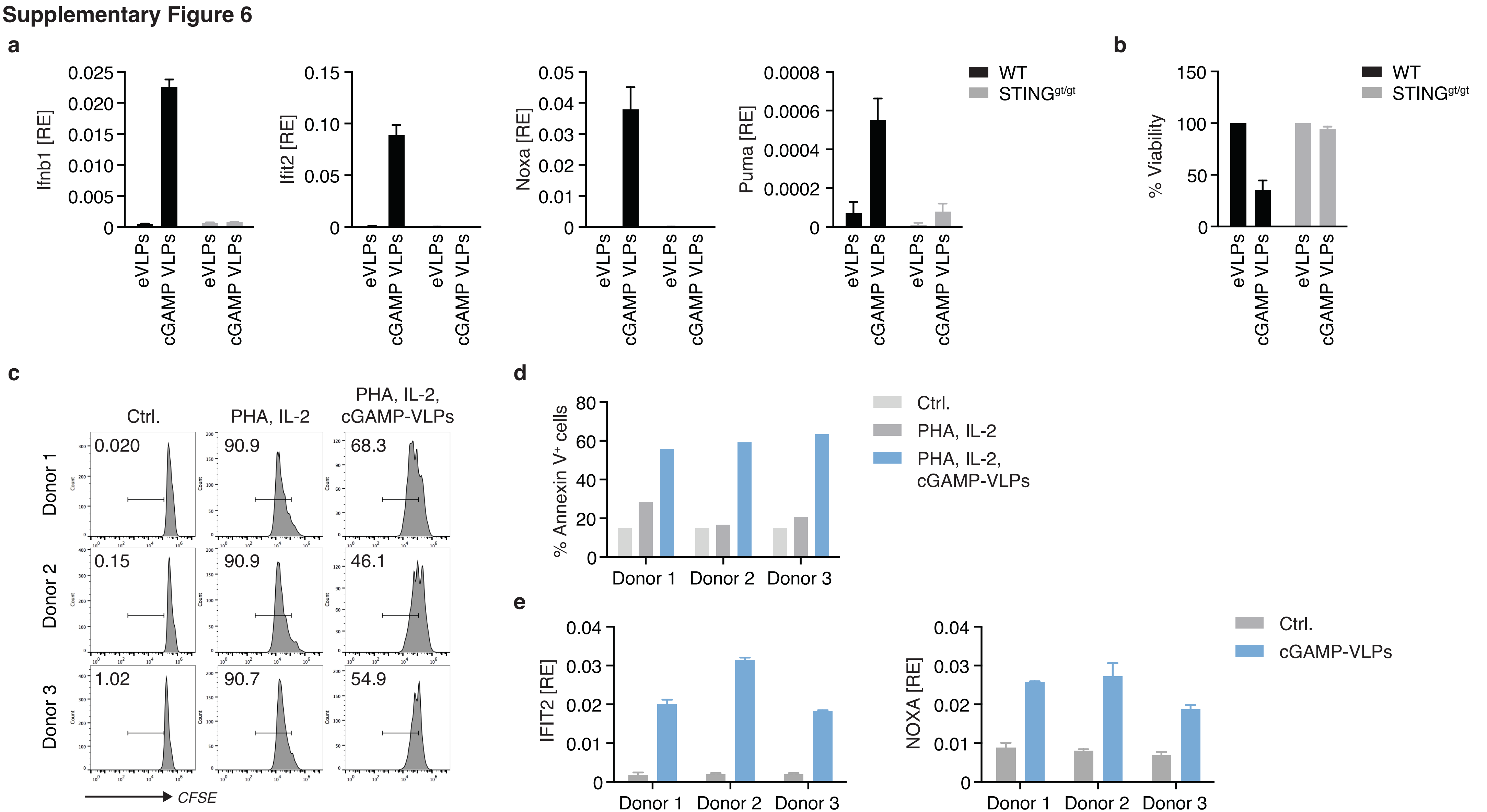

Supplementary Fig. 6 **cGAMP incorporated VLPs induce BH3-only protein expression and cell death in primary T cells**

(**a**, **b**) T cells from WT or STING$^{gt/gt}$ mice were incubated with empty viral particles (eVLPs) or cGAMP containing viral particles (cGAMP VLPs). mRNA abundance of indicated genes was quantified by RT-qPCR after 16 h (**a**) and cell viability was assessed by CellTiter Blue assay after 16 h (**b**). (**c**-**e**) Primary human CD4^{+} T cells from three donors were labeled with CFSE and either activated with PHA-L and IL-2 or cultured in T cell medium. At day 1, cGAMP VLPs were added to the cell culture medium. At day 5 CFSE dilution and Annexin V positive cells were assessed by FACS analysis (**c**, **d**). Human CD4^{+} T cells were expanded with PHA-L and IL-2 for 5 days, incubated with eVLPs or cGAMP VLPs and mRNA abundance of indicated genes was quantified by RT-qPCR after 4 h. Data are representative of three independent experiments (**c**) or mean and s.d. of technical replicates of n=3 independent experiments (**a**, **b**) or mean and s.d. of technical replicates of n=3 distinct donors (**d**, **e**) are shown.

Supplementary Figure 7

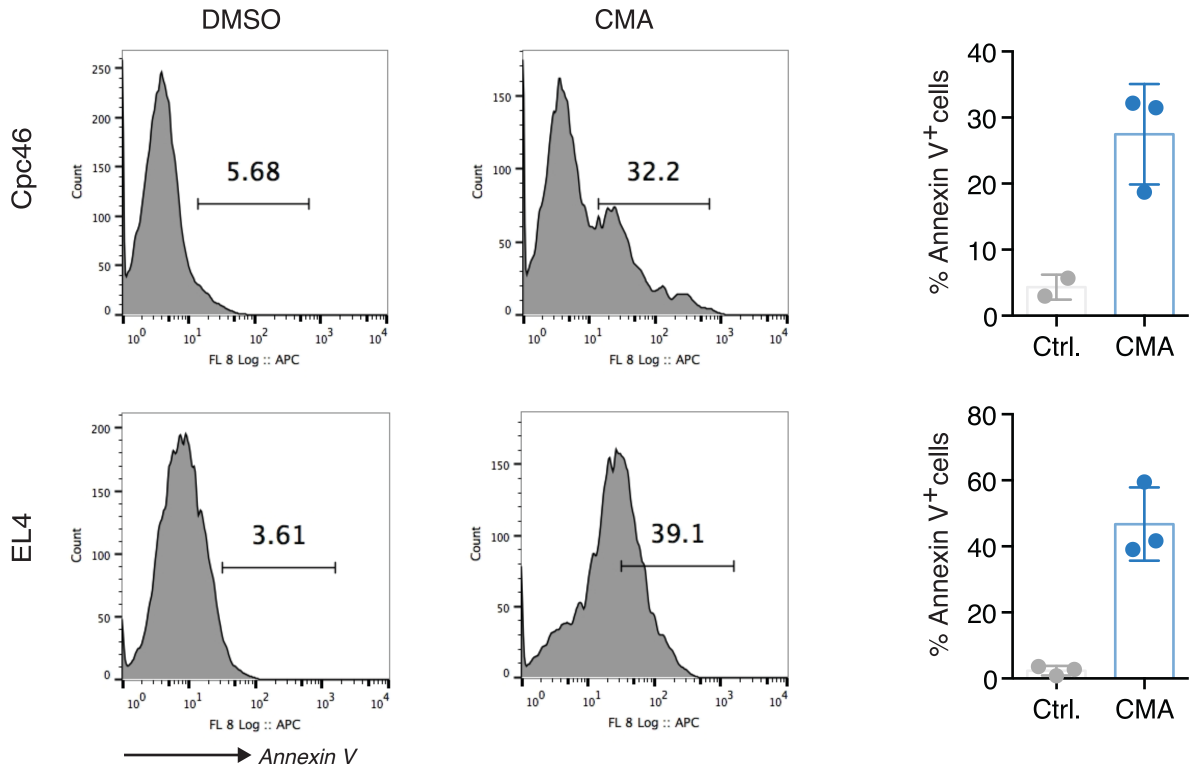

Supplementary Fig. 7 STING activation induces *in vivo* tumor cell apoptosis

Cpc46 cells (upper panel) or EL4 cells (lower panel) were grown in Rag2$^{-/-}$γc$^{-/-}$ mice. After overnight treatment with 10-carboxymethyl-9-acridanone (CMA) (500 μg intratumoral injection) or vehicle (Ctrl., DMSO) tumours were collected and the presence of apoptotic cells was assessed by Annexin V staining via flow cytometry. Left: representative results are depicted; Right: mean and s.d. of percentages of Annexin V positive cells are shown (Cpc46, n = 3 for CMA, n = 2 for ctrl.; EL4, n = 3 per treatment group).

Supplementary Figure 8

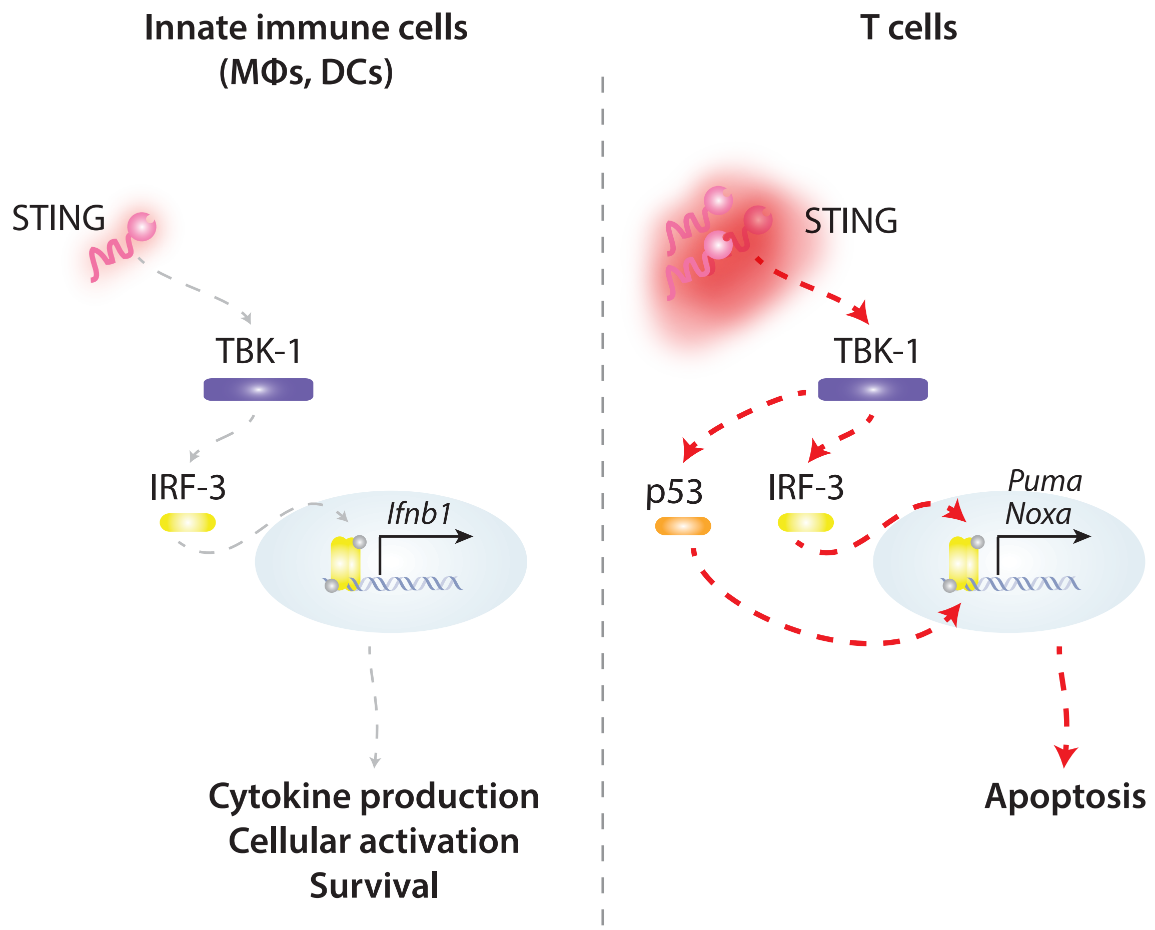

Supplementary Figure 8: Model of cell type-specific responses to STING signaling
Innate immune cells including macrophages (MΦs) and DCs display lower STING expression, thereby generating a balanced response towards STING activators, which leads to cellular activation and cytokine production. T cells express high levels of STING and display an intensified response to STING activators, which leads to apoptosis.

Supplementary Figure 9

Fig. 1a

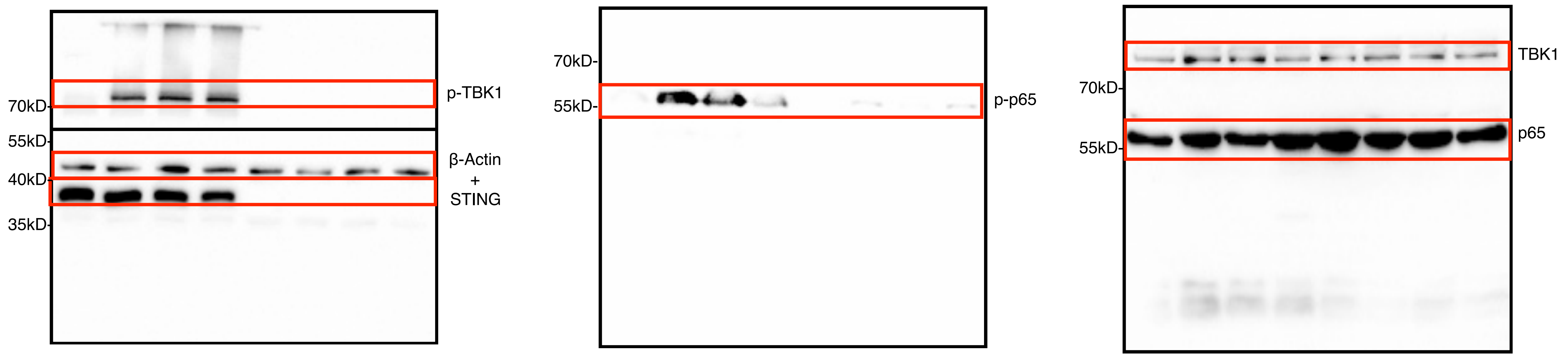

Fig. 1f

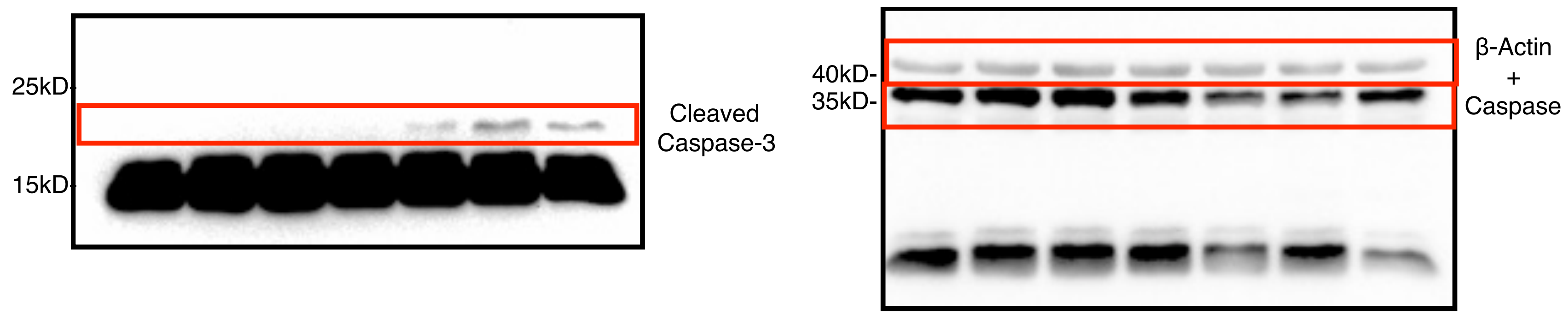

Fig. 2a

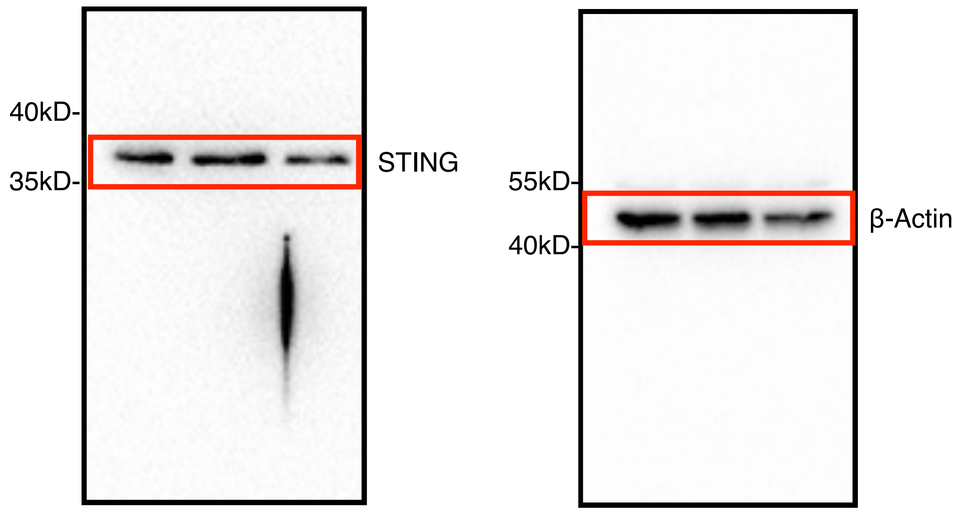

Fig. 4d

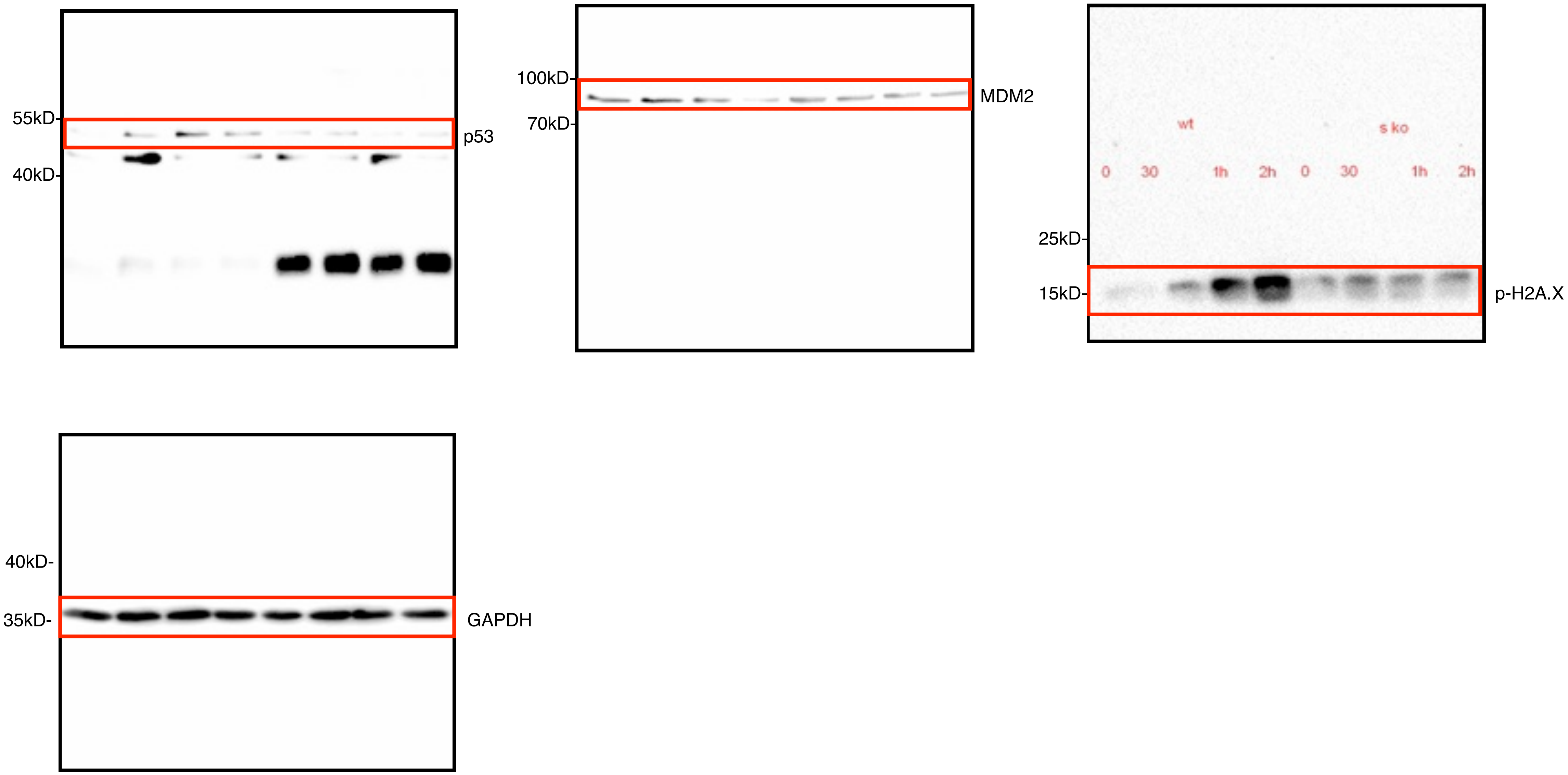
55kD-
40kD-
p53
100kD-
70kD-
MDM2
wt
s ko
0
30
1h
2h
0
30
1h
2h
25kD-
15kD-
p-H2A.X
40kD-
35kD-
GAPDH

Fig. 5a

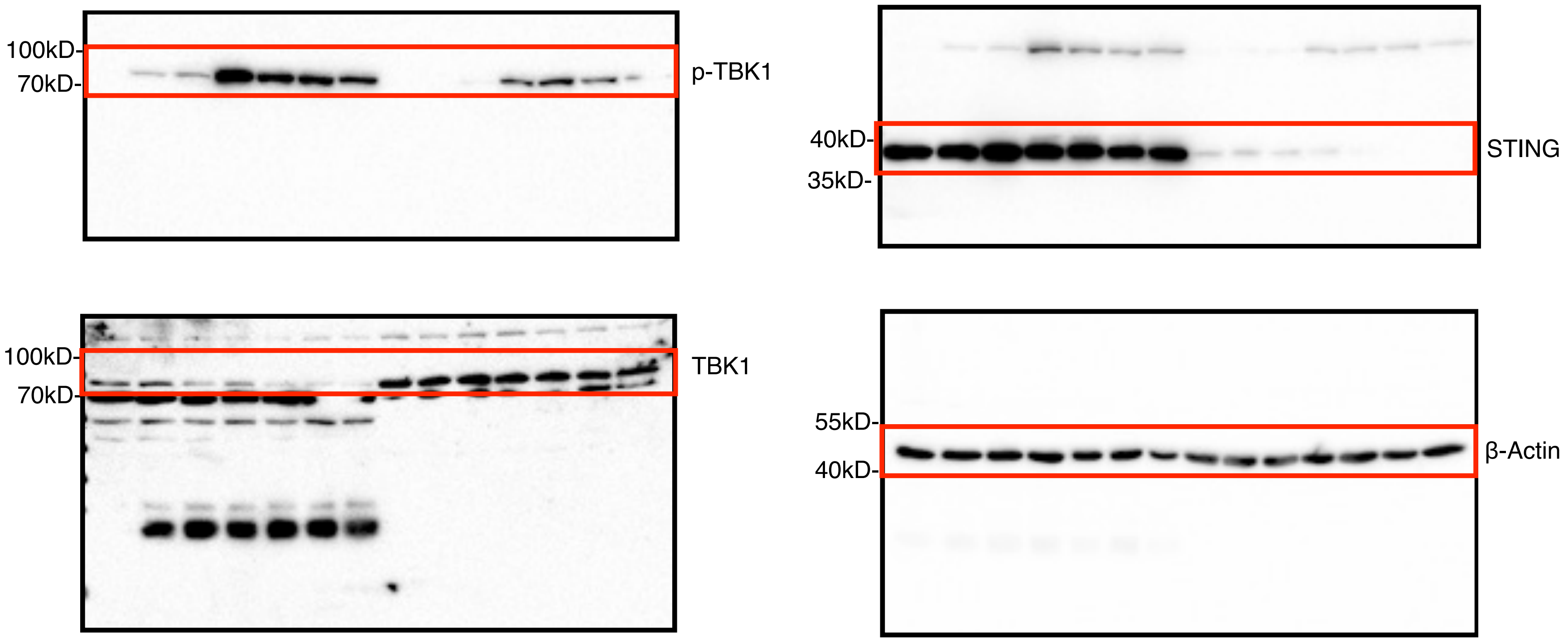
100kD-
70kD-
p-TBK1
40kD-
35kD-
STING
100kD-
70kD-
TBK1
55kD-
40kD-
β-Actin

Fig. 5d

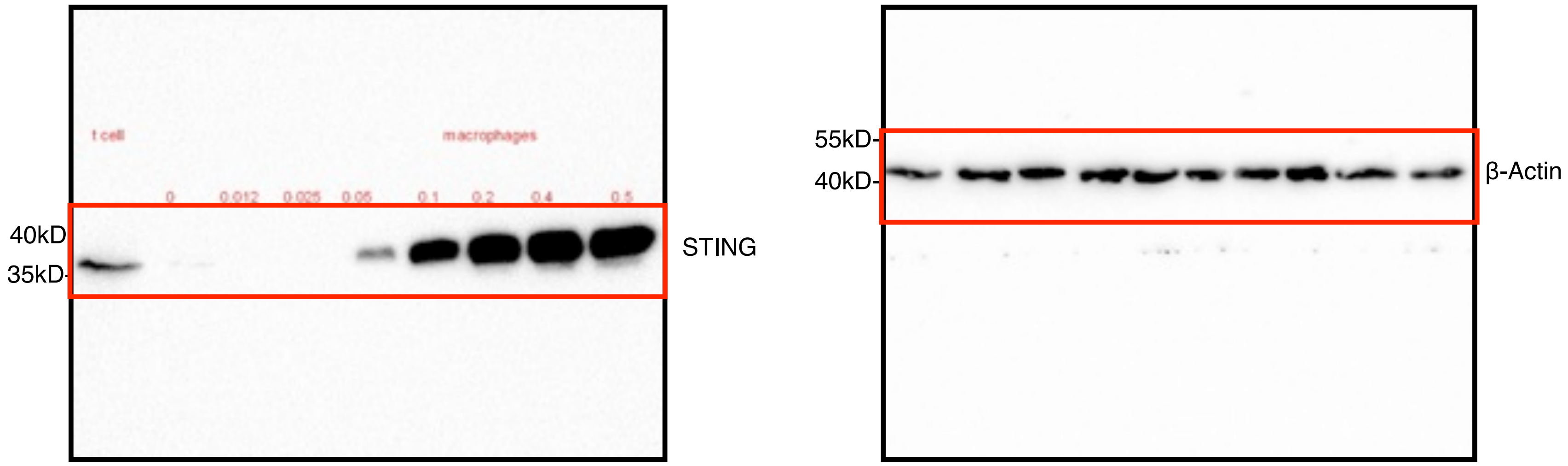
t cell
macrophages
0
0.012
0.025
0.05
0.1
0.2
0.4
0.5
40kD
35kD
STING
55kD-
40kD-
β-Actin

Fig. 6a

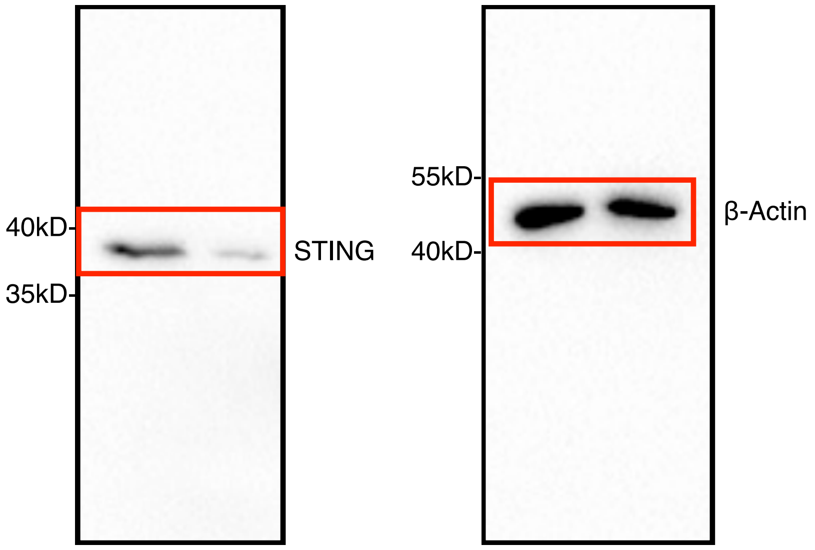

Fig. 6b

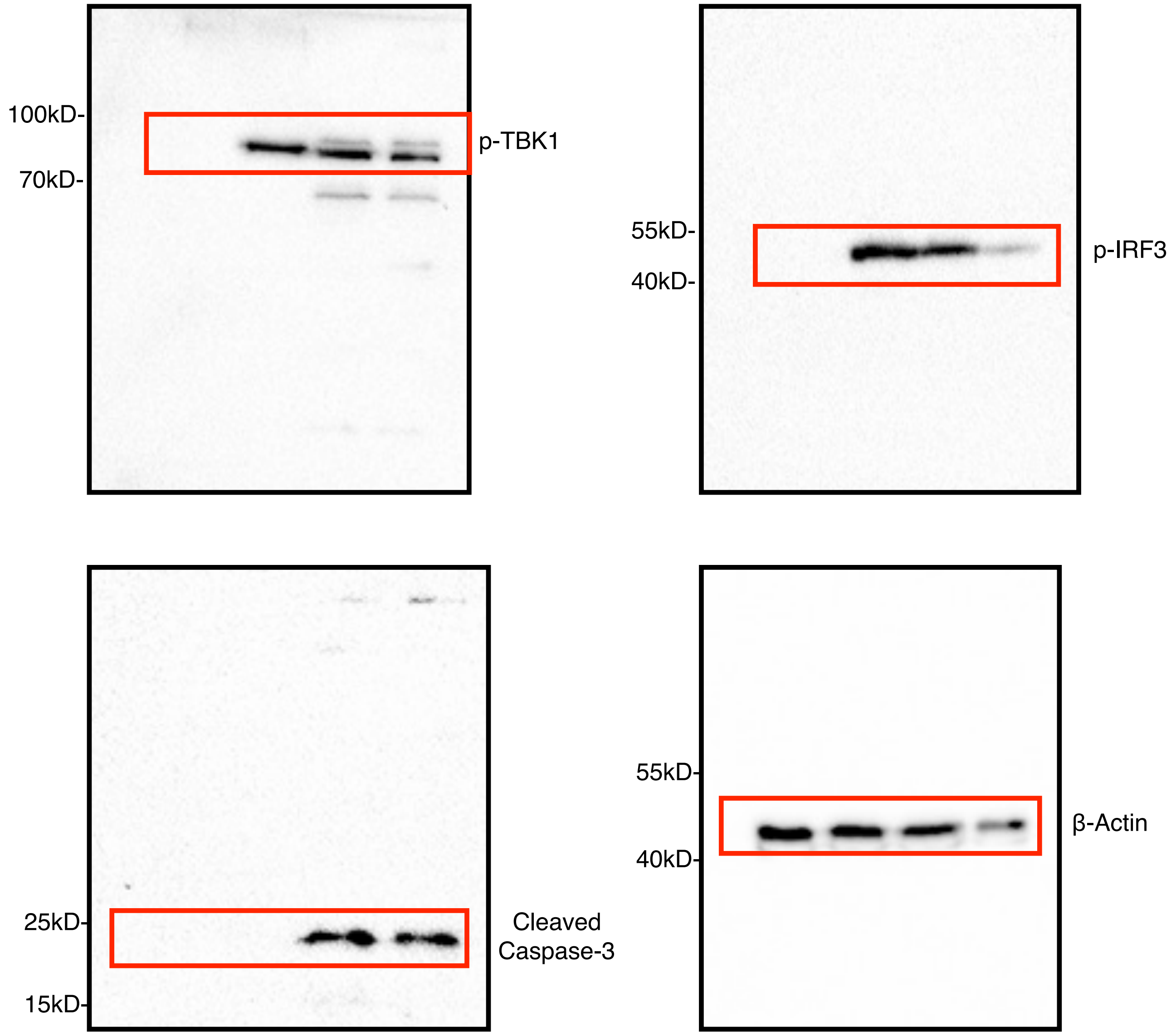

Supplementary Fig. 9 **Uncropped images of western blots**

Supplementary Figure 10

a

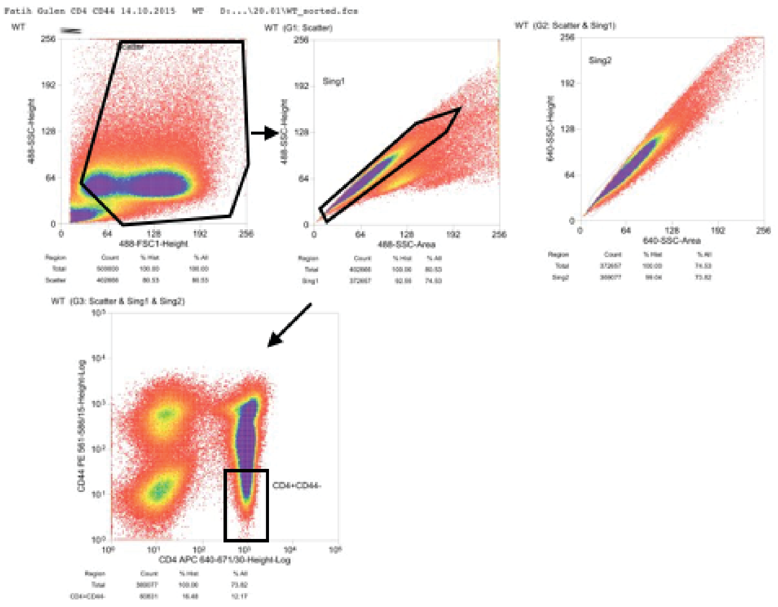

b

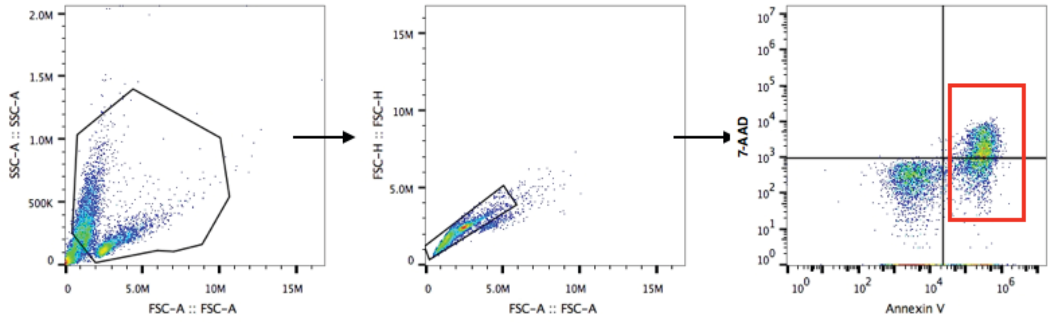

Supplementary Fig. 10 **FACS gating strategy**

(**a**) $CD4^+CD44^-$ cell sorting strategy: Naive T cells were obtained by sorting $CD4^+CD44^-$ cells as shown below in experiments in Fig. 1a, e and Fig. 3a, b. (**b**) Annexin V^+ cell analysis by FACS: Analysis strategy shown below was used to determine the Annexin V^+ population in all experiments including in Fig. 1e; Fig. 3d; Fig. 4a-c and Fig. 6f.
